# Effect of individualized weight management intervention on excessive gestational weight gain and perinatal outcomes: a randomized controlled trial

**DOI:** 10.7717/peerj.13067

**Published:** 2022-03-08

**Authors:** Mei-Yan Xu, Yan-Jun Guo, Li-Juan Zhang, Qing-Bin Lu

**Affiliations:** 1 Department of Nutrition, Aerospace Center Hospital, Beijing, PR China; 2 Department of Obstetrics and Gynecology, Aerospace Center Hospital, Beijing, PR China; 3 Department of Laboratorial Science and Technology, School of Public Health, Peking University, Beijing, PR China

**Keywords:** Individualized weight management, Excessive gestational weight gain, GDM, Hypertension

## Abstract

It is unclear whether weight management is still effective for pregnant women with excessive weight gain in the second or third trimester in China. This study adopted individualized weight management intervention for pregnant women with abnormal weight gain in the second or third trimester, to analyze the effect of intervention by observing the gestational weight gain and perinatal outcomes. This randomized controlled trial was performed at Aerospace Center Hospital. The obstetrician determined whether the pregnant women gained too much weight in the second or third trimester according to the Institute of Medicine guidelines, and randomly divided the pregnant women who gained too much weight in the second or third trimester into the intervention group or the control group according to the inclusion and exclusion criteria. The pregnant women in the intervention group and in the control group all received routine prenatal examination and diet nutrition education by the doctors in the Department of Obstetrics and Gynecology. The intervention group underwent individualized weight management, including individualized diet, exercise, psychological assessment, cognitive intervention and continuous communication, the whole process is tracked and managed by professional nutritionists. The obstetrician collected the prenatal examination data and pregnancy outcome data of all enrolled pregnant women. The primary outcome measure was weight gain during pregnancy. A generalized linear model and a logistic regression model were used to compare the outcomes between the two groups. In total, 348 pregnant women participated in this study with 203 in the intervention group and 145 in the control group. The whole gestational weight gain in the intervention group (15.8 ± 5.4 Kg) was lower than that in the control group (17.5 ± 3.6 Kg; adjusted *β* =  − 1.644; 95% CI [−2.660–−0.627]; *P* = 0.002). The percent of pregnant women with excessive weight gainbefore delivery was 54.2% (110/203) in the intervention group, which was lower than 69.7% (101/145) in the control group (adjusted RR = 0.468; 95% CI [0.284–0.769] *P* = 0.003). The pregnant women given the individualized weight management intervention from the second to the third trimester experienced less weight gain than that from the third trimester (15.5 ± 5.6 Kg *vs.* 16.2 ± 5.2 Kg), but without significant difference (*P* = 0.338). Lower rates of GDM, preeclampsia and gestational hypertension, higher rates of fetal distress and puerperal infection were observed in the intervention group than in the control group (all *P* < 0.05). Individualized weight management during the second or third trimesters is still beneficial for pregnant women who gain excessive weight and can decrease the associated adverse outcomes.

## Introduction

Worldwide, about 50% women of childbearing age are overweight or obese (Branum, Kirmeyer & Gregory, 2016; Flegal et al., 2016). Excessive weight gain during pregnancy for the women of childbearing age is associated with adverse outcomes, including large for gestational age (LGA), macrosomia, a necessary caesarean delivery, gestational diabetes mellitus (GDM), preeclampsia, and offspring obesity (Goldstein et al., 2017; Hannaford et al., 2017; Hedderson, Gunderson & Ferrara, 2010; Mourtakos et al., 2017; Subhan et al., 2017). In China, excessive weight gain occurred in more than 50% pregnant women (Huang et al., 2016).

Women with a high pre-pregnancy body mass index (BMI) have an increased risk for adverse maternal and neonatal outcomes, and gestational weight gain has different effects on the adverse pregnancy outcomes based on the different pre-pregnancy BMI categories (Hung & Hsieh, 2016; Subhan et al., 2017; Widen et al., 2015). Women should be encouraged to enter pregnancy with a healthy BMI and adhere to the current gestational weight gain recommendations (Davies et al., 2010). A retrospective cohort study in Taiwan reported that the fewest maternal and perinatal complications were observed in women whose pregnancy weight gain fell within the range recommended by the Institute of Medicine (IOM) in 2009 (*i.e.,* a total weight gain >11.5 Kg was considered excessive in overweight women and >9 Kg as excessive in obese women for full term, singleton pregnancies) (Horng et al., 2018). One meta-analysis including 36 randomized clinical trials (RCTs) with 12526 participants demonstrated interventions based on diet and physical activity during pregnancy reduced gestational weight gain and lowered the odds of caesarean section (International Weight Management in Pregnancy, i-WIP)Collaborative Group(2017). Another meta-analysis including 49 RCTs with 11,444 participants also reported that diet or exercise, or both, during pregnancy also induced the risk of macrosomia and neonatal respiratory morbidity, particularly for women receiving combined diet and exercise interventions with high-risk (Muktabhant et al., 2015). A two-arm RCT among African–American women reported that a behavioral intervention to promote weight control during pregnancy reduced the proportion of women (from 66% to 37%) who exceeded the IOM guidelines and reduced the amount of weight gain compared to standard care (Herring et al., 2016). Furthermore, a meta-analysis revealed that diet and physical activity interventions designed to reduce gestational weight gain are more effective than standard care in decreasing the incidence of GDM (Bennett et al., 2018).

However, only a few studies have examined the effect of intervention strategies on reducing excessive gestational weight gain in China. A cohort study confirmed that physical activity decreased excessive gestational weight gain either in the second trimester or the third trimester (Jiang et al., 2012). Jing et al. (2015) performed a randomized controlled trial and found that a personalized intervention for weight gain using physical activity during the first trimester significantly lowered the mean weight gain and the rate of GDM. Luo, Dong & Zhou (2014) also found that a nutritional management intervention from the first trimester to delivery prevented excessive gestational weight gain and improved perinatal outcomes. Previous studies mainly focused on the women on the early stage of pregnancy, but not on the pregnancy women with excessive weight gain during the second or third trimester. Whether these interventions are beneficial for pregnancy women with excessive weight gain during the second or third trimester in China remains unclear.

Thus, the aim of this study was to evaluate the effect of individualized weight management intervention during the second and third trimesters for pregnancy women with excessive weight gain by observing the gestational weight gain and perinatal outcomes.

## Material and Methods

### Trial design

This randomized controlled trial studied pregnant women with excessive gestational weight gain at the Aerospace Center Hospital and was designed by the nutritionist and doctors in the Department of Obstetrics and Gynecology. This study was approved by the Ethical Committees of the Aerospace Center Hospital (20170329-YNQN-08) and was registered (ChiCTR1800016876, Chinese Clinical Trial Registry, 2018-6-23). The methods were carried out according to the approved guidelines. All study participants provided informed written consent prior to study enrolment.

### Participants

The inclusion criteria were that pregnant women with excessive gestational weight gain according to the IOM guidelines (Institute of Medicine, 2009) were selected from outpatient services of the Aerospace Center Hospital; all participants were singleton pregnancy, no complications before pregnancy (*i.e.,* type 2 diabetes mellitus, pre-pregnancy hypertension, and renal, immunologic, or hepatic diseases), signed the informed consent form, and all women intended to receive prenatal care and complete the pregnancy at our institution. The exclusion criteria were that pregnancy outcome data were not available. All the participants were provided standard obstetrical care and nutrition education.

### Intervention

The aim of the intervention was to build the study participants’ motivation, support, and self-efficacy for weight-related behavior change based on the Social Cognitive Theory. The procedures of the intervention were as follows. First, the participants were educated on the importance of weight management during pregnancy from the second trimester to the third trimester and the adverse outcomes caused by excessive gestational weight gain by the obstetricians. Second, the participants’ dietary habits were assessed and instructed by nutritionists. All participants in the intervention group were given a dietary habit questionnaire and a 3-day 24-hour dietary survey. Based on the results of the questionnaire, the pregnant women were preliminarily instructed to adjust their dietary structure, indicate the types of food they need to limit or increase, and guide the lifestyle adjustment of eating speed, drinking frequency, and work and rest time. Third, individual meal plans were given by nutritionists. Body composition analysis was performed for each pregnant woman to determine basal metabolic rate. Generally, the initial target energy of pregnant women is basal metabolism plus 300–500 kcal, and then fine-tune the daily target energy and food amount according to the patient’s body fat rate, weight, exercise, dietary habits and so on. According to the 2016 Dietary Guidelines for Pregnant Women in China, it is recommended to make a one-week diet for pregnant women to reference. All participants in the intervention group were asked to keep a food diary for at least five to seven days a month to evaluate the implementation of the dietary intervention and as a basis for the next dietary adjustment. An on-call nutritionist was available to assist the participants with proper nutrient and energy intake. Fourth, the participants were prescribed physical activity according to the American College of Obstetricians and Gynecologists (ACOG) recommendations for at least 150 min per week of moderate-intensity aerobic activity (American College of Obstetricians and Gynecologists, 2015) or at least walking 5000 steps daily until delivery. The participants were required to record the number of daily exercise steps for 5–7 days every month to evaluate the daily exercise status. The participants were asked to weigh themselves weekly with the goal of meeting target IOM weight in the second and third trimesters. During the participant’s check-ups, all dietary and exercise plans were assessed and readjusted based on the participants’ progress. The nutritionist was also available for counselling during the entire study. The participants accepted the intervention from the time of inclusion. Standard dietary advice and precautions were given to the intervention group and the control group by the doctors in the Department of Obstetrics and Gynecology.

### Outcomes

The primary outcome measure was gestational weight gain during pregnancy, that was the difference between the weight on the day of delivery and the weight before pregnancy.

The secondary outcomes were the neonatal outcomes (including birth weight, asphyxia neonatorum, prematurity, fetal macrosomia, fetal distress and the Apgar score at one and five minutes) and the maternal outcomes (including delivery mode, GDM, premature rupture of membrane, postpartum hemorrhage, preeclampsia, gestational hypertension, thyroid diseases, anemia, uterine inertia, abnormal amniotic fluid, and puerperal infection), which were retrieved from the inpatient hospital records after delivery by the obstetrician.

We collected pre-pregnancy information by the doctors in obstetrics department which were self-reported by the pregnant women such as age, gravidity and parity, and the information at birth (the weight, gestation in weeks, and number of hospitalization days). The pre-pregnancy height and weight were defined as the height and weight at the 4th-6th week after pregnancy when establishing the records for the pregnant women, which were measured by the nurse in the gynecological clinic. GDM was diagnosed using a 75-g, 2-hour OGTT as proposed by the International Association of Diabetes and Pregnancy Study Group (International Association of Diabetes et al., 2010) at the 24 gestational weeks. Pregnant women with hypothyroidism were identified according to the ACOG guidelines for thyroid disease during pregnancy (American College of Obstetricians and Gynecologists, 2015).

### Sample size

The sample size was determined to be at least 176 in the intervention group and 117 in the control group. A 95% power value was used to detect a difference of −2.0 between the null hypothesis that both groups’ means were 15.0 and the alternative hypothesis that the mean of control group was 17.0. The estimated group standard deviations were 5.0 and 4.0 for the intervention and control groups, which were estimated by the obstetrician and gynecologist based on previous data on the pregnant women, respectively, and the significance level (alpha) was set at 0.050 using a two-sided two-sample *t*-test.

### Randomization and blinding

Each participant was randomly placed into the intervention group or the control group according to the group result produced by a statistician using a computer in the clinic room. The randomization code was assigned to each participant in sequence in the order of enrolment, and then the participants were assigned to the group corresponding to that code. All eligible participants were assigned a random number which was used to identify all procedures performed after the participant s had been randomly grouped. Once a random number had been assigned to one participant, it could not be reassigned to another participant. The intervention group was given an individualized weight management intervention without blinding method at the nutrition clinic of the Aerospace Center Hospital.

### Statistical analysis

The IOM guidelines suggesting the amount of weight to gain during pregnancy according to the pre-pregnancy BMI were used (Table S1) (2009). We calculated total weight gain during the pregnancy. Excess weight gain was defined as the difference between total weight gain during the pregnancy and the upper cutoffs of normal total weight gain at different BMI groups. The study adheres to the CONSORT guidelines. The second trimester was defined as the period from the 13th week to the 27th week and the third trimester was defined as the period from the 28th to birth. Premature delivery was defined as <37 gestational weeks according to the World Health Organization (WHO) classification from 1977.

The BMI was calculated by the weight (Kg) divided by the square of the height (m^2^). Obesity was defined as a BMI equal to or greater than 28 Kg/m^2^, overweight was defined as a BMI equal to or greater than 24 Kg/m^2^ and less than 28 Kg/m^2^, normal weight was defined as a BMI equal to or greater than 18.5 Kg/m^2^ and less than 24 Kg/m^2^ and underweight was defined as a BMI less than 18.5 Kg/m^2^ as recommended by the Working Group on Obesity in China (Zhou & Cooperative Meta-Analysis Group of the Working Group on Obesity in China, 2002).

Descriptive statistics were performed, with continuous variables reported as the mean  ±  standard deviation (SD) for normal distribution and median (interquartile range, IQR) for abnormal distribution and categorical variables reported as frequencies and proportions. Generalized linear models assessed the association between the intervention or intervention time point and the continuous outcome variables, including the prenatal weight of pregnant women, weight gain during pregnancy, prenatal BMI, gain in BMI during pregnancy, newborn birth weight, gestational weeks and length of hospitalization. The *β* and 95% confidence interval (95% CI) were estimated. A logistic regression model was used to explore the rate difference of the categorical outcome variables (including caesarean, premature rupture of membrane, asphyxia neonatorum, prematurity, fetal macrosomia, GDM, postpartum haemorrhage, fetal distress, preeclampsia, gestational hypertension, thyroid diseases, anaemia, uterine inertia, abnormal amniotic fluid, puerperal infection, an Apgar score at one minute of <10 and an Apgar score at five minutes of <10) between the intervention and control groups or between the second and third trimester groups, which is when the participants began the individualized weight management plan. The risk ratio (RR) and 95% CI were estimated. We also analyzed the difference of weight gain between the intervention group and control group in the various of the pre-pregnancy BMI groups or in the second and third trimesters using the generalized linear models. For the two models above, the association was adjusted for age, pre-pregnancy BMI, parity and gravidity. The level of significance was set at 0.05. All statistical analyses were performed using Stata 14.0 (Stata Corp LP, College Station, TX, USA).

## Results

### Characteristics of participants in the intervention and control groups

**Figure 1:**
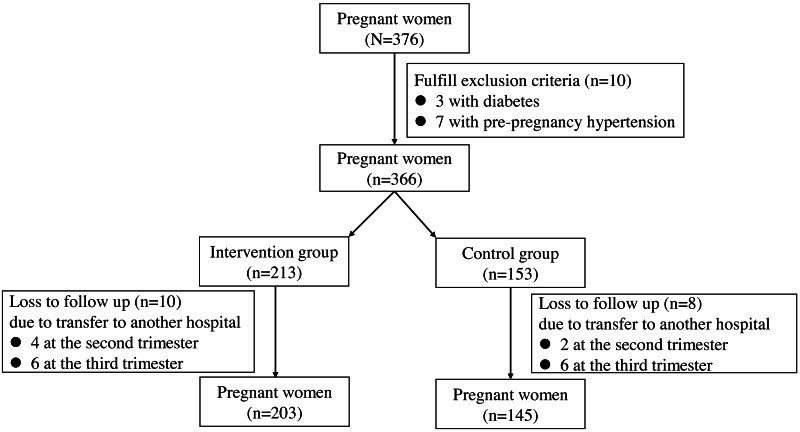
A flow diagram of the pregnant women in the study.

A total of 376 pregnant women were recruited in the study. Among these patients, 28 pregnant women were excluded due to three with diabetes, seven with pre-pregnancy hypertension and 18 loss to follow-up (Fig. 1). Finally, 348 pregnant women participated in the study, with 203 in the intervention group and 145 in the control group. The characteristics of age, height, pre-pregnancy weight, BMI, gravidity and parity were comparable in the two groups (all *P* > 0.05, Table 1). The percent of individuals with obesity was 7.9% and 10.1% in the intervention group and the control group, respectively (*P* = 0.072).

**Table 1:** The characteristics of the participants at baseline in the intervention and control groups.

Variable	Intervention (*n* = 203)	Control (*n* = 145)	*P*
Age, year, mean ± SD	30.3 ± 4.0	30.0 ± 4.2	0.581^*^
Height, cm, mean ± SD	162.8 ± 7.6	161.5 ± 8.6	0.099^*^
Pre-pregnancy weight, Kg, mean ± SD	59.6 ± 10.0	59.8 ± 10.0	0.829^*^
Pre-pregnancy BMI, Kg/m^2^, mean ± SD	22.7 ± 5.4	23.5 ± 11.3	0.317^*^
Underweight	23 (10.8)	23 (11.0)	0.666^#^
Normal	129 (60.6)	115 (55.0)	
Overweight	44 (20.7)	50 (23.9)	
Obese	17 (7.9)	21 (10.1)	
Gravidity			0.723 ^#^
1	98 (46.0)	104 (49.8)	
2	59 (27.7)	55 (26.3)	
3	38 (17.8)	30 (14.4)	
>3	18 (8.5)	20 (9.5)	
Parity			0.202 ^#^
1	146 (68.5)	155 (74.2)	
>1	67 (31.5)	54 (25.8)	

**Notes.**

* *t*-test.

# chi-square test.

SD, standard deviation.

### Pregnant outcomes in the intervention and control groups

We compared the prenatal weight of pregnant women and newborn weight between the two groups (Table 2). No significant differences were found in the prenatal weight of pregnant women after adjusting for age, pre-pregnancy BMI, parity and gravidity (*P* = 0.162). Notably, the gestational weight gain in the intervention group (15.8 ± 5.4 Kg) was lower than that in the control group (17.5 ± 3.6 Kg, adjusted *β* =−1.644; 95%CI: −2.660, −0.627; *P* = 0.002), which was also observed for the BMI gain during pregnancy (*P* = 0.014). The excessive weight gain rate was 54.2% (110/203) in the intervention group, which was lower than that in the control group [69.7% (101/145)] (adjusted RR = 0.468; 95% CI [0.284–0.769]; *P* = 0.003). The newborn babies also had a lower birth weight in the intervention group than in the control group without statistically significance (adjusted *β* =−0.086; 95% CI [−0.181 to −0.010]; *P* = 0.079). The pregnant women in the intervention group had a shorter hospitalization stay compared to the control group (5.0 *vs.* 5.8 days, *P* = 0.001).

**Table 2:** The comparison of weight and BMI in the intervention and control groups.

Variable	Intervention	Control	Adjusted^*^ β (95% CI)	*P*
Prenatal weight of pregnant women, Kg, mean ± SD	75.2 ± 10.5	76.5 ± 10.6	−1.124 (−2.698, 0.450)	0.162
Weight gain during pregnancy, Kg, mean ± SD	15.8 ± 5.4	17.5 ± 3.6	−1.644 (−2.660, −0.627)	0.002
Prenatal BMI, Kg/m^2^, mean ± SD	28.6 ± 6.9	30.1 ± 15.3	−1.112 (−3.256, 1.032)	0.309
BMI gain during pregnancy, Kg/m^2^, mean ±SD	6.1 ± 2.6	6.8 ± 2.5	−0.671 (−1.208, −0.135)	0.014
Newborn birth weight, Kg, mean ± SD	3.38 ± 0.43	3.46 ± 0.46	−0.086 (−0.181, 0.010)	0.079
Gestational weeks, mean ±SD	39 ± 1	39 ± 1	−0.025 (−0.277, 0.226)	0.843
Hospitalization, days, mean ±SD	5.0 ± 1.9	5.8 ± 2.2	−0.708 (−1.128, −0.288)	0.001

**Notes.**

* The generalized linear model was adjusted for age, pre-pregnancy BMI, parity and gravidity.

SD, standard deviation; BMI, body mass index.

**Figure 2:**
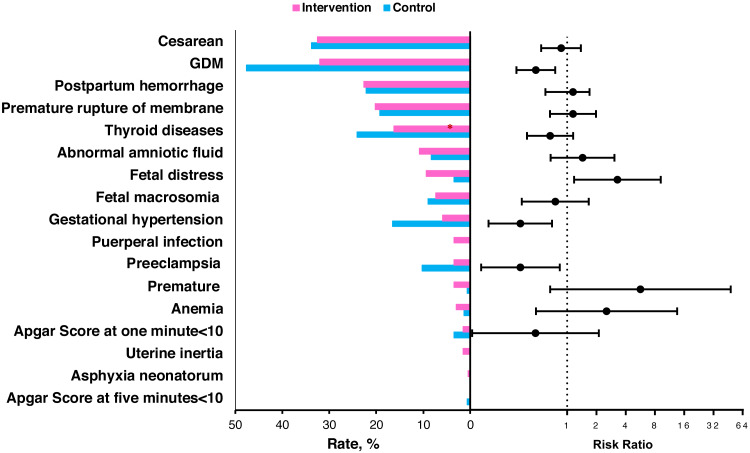
The percent of individuals in the intervention and control groups with the various pregnancy outcomes and the risk ratios of the intervention group for the pregnancy outcomes. The risk ratio and 95% confidence interval were estimated by a logistic regression model when adjusted for age, pre-pregnancy BMI, parity and gravidity. SD, standard deviation; BMI, body mass index.

The rates of three complications were lower in the intervention group than in the control group, including GDM (32.0% *vs.* 47.6%; adjusted RR =0.475; 95%CI [0.30–0.752]; *P* = 0.001), preeclampsia (3.5% *vs.* 10.3%; adjusted RR =0.330; 95%CI [0.130–0.838]; *P* = 0.009) and gestational hypertension (5.9% *vs.* 16.6%; adjusted RR =0.329; 95%CI [0.155–0.693]; *P* = 0.004), as shown in Fig. 2 and Table S2. We also found higher rates of fetal distress (9.4% *vs.* 3.5%, *P* = 0.023) and puerperal infection (3.5% *vs.* 0%, *P* = 0.044 by Fisher exact test) in the intervention group compared to the control group. No significant differences of other complications were found between the two groups.

### Pregnancy outcomes between the second and third trimester groups

**Figure 3:**
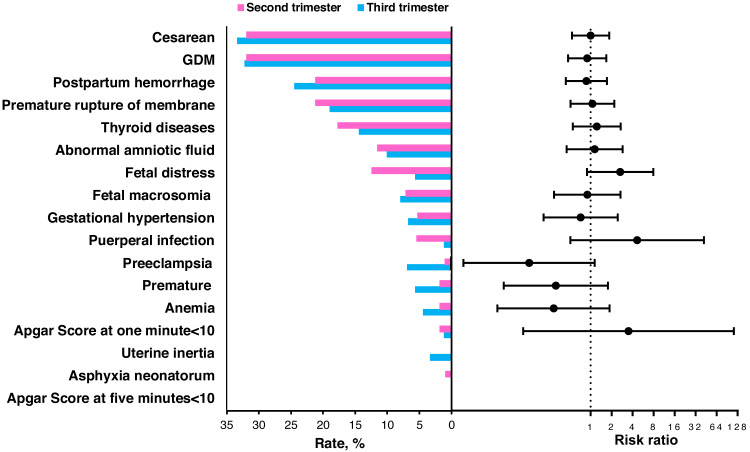
The percent of pregnancy outcomes when starting the intervention during the second and third trimester groups and the risk ratios of the second trimester group compared to the third trimester. The risk ratio and 95% confidence interval were estimated by a logistic regression model and adjusted for age, pre-pregnancy BMI, parity and gravidity. SD, standard deviation; BMI, body mass index.

There were 113 pregnant women who started the intervention from the second trimester and 90 from the third trimester. Between these two groups, most of the characteristics shown in Table 3 were comparable, except for height (*P* = 0.033). The distribution of the gestational week when starting the individualized weight intervention was shown in Fig. S1. The percent of women who gained excessive weight was 50.4% (57/113) in the second trimester, lower than 58.9% (53/90) in the third trimester (adjusted RR =0.656; 95% CI [0.357–1.207]; *P* = 0.176). The pregnant women in the second trimester group gained less weight during pregnancy than those in the third trimester group (15.5 ± 5.6 Kg *vs.* 16.2 ±5.2 Kg), but the difference was not significant (adjusted *β* =−0.739; 95% CI [−2.248–0.771]; *P* = 0.338; Table 4). The percent of participants with preeclampsia from the second trimester group to the third trimester group was 0.9% and 6.7%, respectively, (*P* = 0.066) as shown in Fig. 3 and Table S3.

**Table 3:** The distribution of the participants during the second and third trimester groups in the intervention group.

Variable	Second (*n* = 113)	Third (*n* = 90)	*P*
Age, year, mean ± SD	30.1 ± 3.6	30.2 ± 4.4	0.785^*^
Height, cm, mean ± SD	163.1 ± 9.5	162.5 ± 4.6	0.033^*^
Pre-pregnancy weight, Kg, mean ± SD	59.8 ± 11.0	58.7 ± 8.0	0.655^*^
Pre-pregnancy BMI, Kg/m^2^, mean ± SD	22.8 ± 6.8	22.2 ± 2.9	0.802^*^
Underweight	15 (13.3)	8 (8.9)	0.095^#^
Normal	66 (58.4)	58 (64.4)	
Overweight	21 (18.6)	22 (24.4)	
Obese	11 (9.7)	2 (2.2)	
Gravidity			0.109^#^
1	59 (52.2)	36 (40.0)	
2	22 (19.5)	31 (34.4)	
3	22 (19.5)	16 (17.8)	
>3	10 (8.8)	7 (7.8)	
Parity			0.160^#^
1	82 (72.6)	57 (63.3)	
>1	31 (27.4)	33 (36.7)	

**Notes.**

* *t*-test.

# chi-square test.

**Table 4:** The comparison of weight and BMI during the second and third trimester groups in the intervention group.

Variable	Second (*n* = 113)	Third (*n* = 90)	Adjusted^*^ β (95% CI)	*P*
Prenatal weight of pregnant women, Kg, mean ± SD	75.3 ± 11.2	75.0 ± 9.5	−0.192 (−2.395, 2.012)	0.865
Weight gain during pregnancy, Kg, mean ± SD	15.5 ± 5.6	16.2 ± 5.2	−0.739 (−2.248, 0.771)	0.338
Prenatal BMI, Kg/m^2^, mean ±SD	28.8 ± 8.6	28.4 ± 3.6	−0.367 (−1.941, 1.208)	0.648
BMI gain during pregnancy, Kg/m^2^, mean ± SD	6.0 ± 2.9	6.2 ± 2.0	−0.331 (−1.043, 0.382)	0.363
Newborn birth weight, Kg, mean ± SD	3.39 ± 0.39	3.38 ± 0.48	0.005 (−0.119, 0.128)	0.941
Gestational weeks, mean ± SD	39 ± 1	39 ± 1	0.420 (0.067, 0.773)	0.020
Hospitalization, days, mean ± SD	5.2 ± 2.2	4.8 ± 1.4	0.380 (−0.151, 0.912)	0.160

**Notes.**

* The generalized linear model was adjusted for age, pre-pregnancy BMI, parity and gravidity.

SD, standard deviation; BMI, body mass index.

### Weight gain during pregnancy in the various pre-pregnancy BMI groups

**Figure 4:**
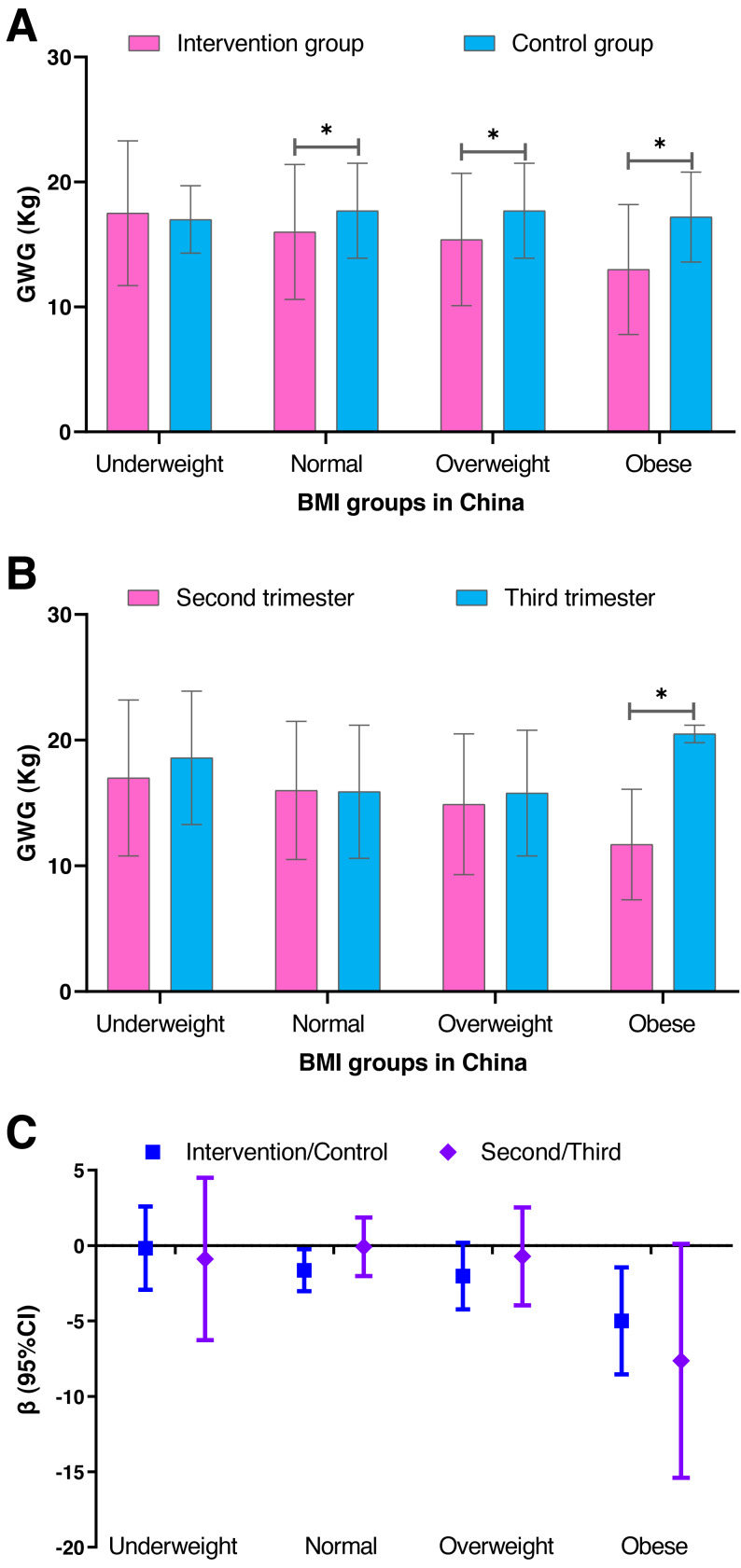
A comparison of the gestational weight gain between the intervention and control groups and between the second and third trimester groups within the various BMI groups in China. (A) Comparison of the gestational weight gain between the intervention and control groups. (B) Comparison of the gestational weight gain between the second and third trimester groups. (C) The *β* and 95% confidence interval were estimated by the generalized regression model and adjusted for age, pre-pregnancy BMI, parity and gravidity. SD, standard deviation; BMI, body mass index; GWG, gestational weight gain. The blue represents the intervention and control groups, and the pink represents the second and third trimester groups.

We performed a stratification analysis based on the pre-pregnancy BMI categories to further observe the effect of individualized weight management on gestational weight gain (Fig. 4 and Table S4). The difference in weight gain between the intervention group and the control group increased as the BMI group increased, especially for the obese group (adjusted *β* =−4.995; 95% CI [−8.542–−1.448]; *P* = 0.006). However, the difference in the weight gain between the second and third trimester groups was approximately the same in the underweight, normal and overweight groups (Fig. 4 and Table S5). The weight gain was less, but not significant (*P* = 0.053), when starting the individualized weight management in the third trimester (adjusted *β* =−7.641; 95% CI [−15.394–−0.111]).

## Discussion

We evaluated the effect of individualized weight management therapies on the pregnant women with excessive gestational weight gain and found that the intervention was useful to decrease weight gain and certain adverse outcomes, especially in the pregnant women who were pre-pregnancy obese. The intervention maybe work better from the second trimester than from the third trimester.

A study in African American women reported that the intervention participants gained less weight during their pregnancy with an adjusted mean difference of −3.1 Kg (Herring et al., 2016), which is greater than our study (−1.64 Kg). Additionally, the percent of participants with excessive weight gain in the aforementioned study (29%) was also greater than our study (15%), which may be related to several factors, including the economic status of the pregnant women, lifestyle differences, compliance to the intervention strategy, and the time point when the intervention began. Jiang et al. (2012) also found that the active group in their study had 1.1 Kg and 1.4 Kg less gestational weight gain during the second and third trimester, respectively, than the sedentary group. Another study evaluated the effects of personalized intervention on weight gain and physical activity during the first trimester and found that the total gestational weight gain in the intervention group was slightly lower than in the control group. Luo et al. provided nutritional management from the first trimester and found that the body weight of the intervention group was approximately 5 Kg less than the control group at 37∼40 weeks (Luo, Dong & Zhou, 2014). Primarily, we found that reducing the gestational weight gain using nutritional intervention and/or physical activity was beneficial. In our study, the key of effective intervention lies in the improvement of pregnant women’s compliance through the co-work of obstetricians, nutritionists and family members. The obstetricians and gynecologists give the detailed introduction on the risks of excessive gestational weight gain. The nutritionists give specific methods and advice on preventing excessive gestational weight gain. Her husband or her families provide family support to implement the individualized weight management intervention plan.

Due to the various populations studied, the intervention methods, and the intervention initiation time point in the aforementioned studies, comparing the intervention effect among these studies is difficult. In our study, although the pregnancy outcome rates were not significant between the second and third trimester groups, most of the outcome rates in the second trimester group were lower than those in the third trimester group. Considering the small sample size, therefore, whether an earlier intervention provides better outcomes needs to be further studied.

In our study, the intervention affected the various pre-pregnancy BMI groups differently. The reduction of weight gain increased as the BMI group increased. Additionally, for the pre-pregnancy obese group, the weight gain was less when the intervention began during the second trimester than during the third trimester. However, no differences were observed in the other three BMI groups, which may be due to compliance of the pregnant women and the importance they placed on the intervention. The obese women (as determined by the pre-pregnancy BMI) may have recognized and accepted the viewpoint that excess gestational weight gain could adversely affect the pregnancy outcomes and therefore better complied with the intervention during pregnancy.

 Ruhstaller et al. (2016) found that women whose weight gain exceeded the IOM guidelines were 1.7 times more likely to develop hypertension during the second trimester and obese women had a 2.4-fold increased risk of developing hypertension. In our study, the pregnant women in the intervention group gained less gestational weight and had a lower rate of gestational hypertension. Additionally, excessive pregnancy weight gain also increases the risk of GDM, resulting in deleterious effects on the pregnancy outcomes (Brunner et al., 2015). Therefore, avoiding excessive pregnancy weight gain may be a valuable solution to reduce GDM, and this hypothesis was confirmed in our study as the intervention group had a lower rate of GDM compared to the control group (32.0% *vs.* 47.6%, respectively). Jiang et al. also observed similar results (22.6% *vs.* 34.9%) (Jing et al., 2015).

When interpreting these results of our study, there were some limitations to consider. First, our results were from women living in urban Beijing, and thus, this study does not comprehensively represent women in rural areas. Second, we did not measure the pre-pregnancy weight and height of the participants in our study, which maybe have bias on calculating the primary outcome. As there are no standard values for gestational weight gain in China, we used the same IOM guidelines related to the BMI categories definitions in China, which may have a bias on the results. Finally, the sample size was not large enough to compare the group starting the intervention from the second trimester with the group starting from the third trimester. We also did not collect any process evaluation data or information on participant compliance, which may be related to the effect of the intervention.

## Conclusion

This study indicates that individualized weight management starting from the second trimester, and even from the third trimester, is useful to control gestational weight gain and decrease the associated adverse outcomes. Gestational weight management should be brought into routine prenatal care to improve the quality of obstetric care and decrease the incidence of maternal and fetal diseases.

##  Supplemental Information

10.7717/peerj.13067/supp-1 Supplemental Information 1 Raw data 28 original variables

10.7717/peerj.13067/supp-2 Supplemental Information 2 New recommendations for total and rate of weight gain during pregnancy by prepregnancy BMI

10.7717/peerj.13067/supp-3 Supplemental Information 3 The outcomes of pregnancy in the intervention and control groups

10.7717/peerj.13067/supp-4 Supplemental Information 4 The outcomes of pregnancy during the second and third trimester groups in the intervention group

10.7717/peerj.13067/supp-5 Supplemental Information 5 The comparison of weight gain of pregnancy women during pregnancy in the various BMI groups between the intervention and control groups and between during the second and third trimester groups

10.7717/peerj.13067/supp-6 Supplemental Information 6 The comparison of weight gain of pregnancy women during pregnancy in the various BMI groups between during the second and third trimester groups

10.7717/peerj.13067/supp-7 Supplemental Information 7 The gestational weeks of the pregnant women in the intervention group. The blue represents the second trimester, and the pink represents the third trimester The gestational weeks of the pregnant women in the intervention group. The blue represents the second trimester, and the pink represents the third trimester.

10.7717/peerj.13067/supp-8 Supplemental Information 8 CONSORT Checklist

10.7717/peerj.13067/supp-9 Supplemental Information 9 Trial protocol

## Abbreviations

LGA: Large for gestational age
GDM: Gestational diabetes mellitus
BMI: Body mass index
IOM: Institute of Medicine
ACOG: American College of Obstetricians and Gynecologists
WHO: World Health Organization
SD: Standard deviation
RR: Risk ratio
CI: Confidence interval

## Additional Information and Declarations

### Data Availability

The following information was supplied regarding data availability: The raw data is available in the Supplemental File.

### Clinical Trial Registration

The following information was supplied regarding Clinical Trial registration: ChiCTR1800016876.
