# Supplementary material for: Effect of individualized weight management intervention on excessive gestational weight gain and perinatal outcomes: a randomized controlled trial
Source: PeerJ. 2022 Mar 8;10:e13067. doi: 10.7717/peerj.13067 (PMC8916027; doi:10.7717/peerj.13067)
Supplement: Supplemental Information 2 [file peerj-10-13067-s002.docx]

Supplemental Table 1. Recommendations for total and rate of weight gain during pregnancy by pre-pregnancy BMI from IOM 2009.

| Pre-pregnancy BMI* | Total weight gain in kg | Mean (range) weight gain in kg/week |
| --- | --- | --- |
| Underweight | 12.5-18 | 0.51 (0.44-0.58) |
| Normal weight | 11.5-16 | 0.42 (0.35-0.50) |
| Overweight | 7-11.5 | 0.28 (0.23-0.33) |
| Obese | 5-9 | 0.22 (0.17-0.27) |

Calculations assume a 0.5–2 kg weight gain in the first trimester.

*The BMI groups was classified by WHO.
