# Supplementary material for: Effect of individualized weight management intervention on excessive gestational weight gain and perinatal outcomes: a randomized controlled trial
Source: PeerJ. 2022 Mar 8;10:e13067. doi: 10.7717/peerj.13067 (PMC8916027; doi:10.7717/peerj.13067)
Supplement: Supplemental Information 3 [file peerj-10-13067-s003.docx]

Supplemental Table 2. The outcomes of pregnancy in the intervention and control groups

| **Outcome** | **Intervention**  **n (%)** | **Control**  **n (%)** | **RR (95%CI)^#^** | **P** |
| --- | --- | --- | --- | --- |
| Cesarean | 66 (32.5) | 49 (33.8) | 0.865 (0.540-1.384) | 0.544 |
| Premature rupture of membrane | 41 (20.2) | 28 (19.3) | 1.146 (0.664-1.979) | 0.624 |
| Asphyxia neonatorum | 1 (0.5) | 0 | - | 1.000^*^ |
| Premature | 7 (3.5) | 1 (0.7) | 5.669 (0.667-48.151) | 0.112 |
| Fetal macrosomia | 15 (7.4) | 13 (9.0) | 0.754 (0.341-1.667) | 0.486 |
| GDM | 65 (32.0) | 69 (47.6) | 0.475 (0.300-0.752) | 0.001 |
| Postpartum hemorrhage | 46 (22.7) | 32 (22.2) | 1.004 (0.596-1.694) | 0.987 |
| Fetal distress | 19 (9.4) | 5 (3.5) | 3.285 (1.179-9.154) | 0.023 |
| Preeclampsia | 7 (3.5) | 15 (10.3) | 0.330 (0.130-0.838) | 0.009 |
| Gestational hypertension | 12 (5.9) | 24 (16.6) | 0.329 (0.155-0.697) | 0.004 |
| Thyroid diseases | 33 (16.3) | 35 (24.1) | 0.667 (0.386-1.153) | 0.147 |
| Anemia | 6 (3.0) | 2 (1.4) | 2.540 (0.477-13.514) | 0.477 |
| Uterine inertia | 3 (1.5) | 0 | - | 0.269^*^ |
| Abnormal amniotic fluid | 22 (10.8) | 12 (8.3) | 1.438 (0.676-3.057) | 0.428 |
| Puerperal infection | 7 (3.5) | 0 | - | 0.044^*^ |
| Apgar Score at one minute <10 | 3 (1.5) | 5 (3.5) | 0.471 (0.105-2.116) | 0.326 |
| Apgar Score at five minutes<10 | 0 | 1 (0.7) | - | 0.417^*^ |

*Fisher exact test. #The generalized linear model was adjusted by the variables of age, BMI before pregnancy, parity and gravidity.
