# Supplementary material for: Effect of individualized weight management intervention on excessive gestational weight gain and perinatal outcomes: a randomized controlled trial
Source: PeerJ. 2022 Mar 8;10:e13067. doi: 10.7717/peerj.13067 (PMC8916027; doi:10.7717/peerj.13067)
Supplement: Supplemental Information 4 [file peerj-10-13067-s004.docx]

Supplemental Table 3. The outcomes of pregnancy during the second and third trimester groups in the intervention group

| **Outcome** | **Second**  **n (%)** | **Third**  **n (%)** | **RR (95%CI)^#^** | **P** |
| --- | --- | --- | --- | --- |
| Cesarean | 36 (31.9) | 30 (33.3) | 0.999 (0.540-1.847) | 0.998 |
| Premature rupture of membrane | 24 (21.2) | 17 (18.9) | 1.059 (0.515-2.180) | 0.876 |
| Asphyxia neonatorum | 1 (0.9) | 0 | - | 1.000^*^ |
| Premature | 1 (0.9) | 5 (5.6) | 0.318 (0.057-1.771) | 0.142^*^ |
| Fetal macrosomia | 8 (7.1) | 7 (7.9) | 0.895 (0.299-2.684) | 0.843 |
| GDM | 36 (31.9) | 29 (32.2) | 0.893 (0.474-1.681) | 0.726 |
| Postpartum hemorrhage | 24 (21.2) | 22 (24.4) | 0.869 (0.440-1.714) | 0.685 |
| Fetal distress | 14 (12.4) | 5 (5.6) | 2.646 (0.887-7.898) | 0.081 |
| Preeclampsia | 1 (0.9) | 6 (6.7) | 0.132 (0.015-1.143) | 0.066 |
| Gestational hypertension | 6 (5.3) | 6 (6.7) | 0.719 (0.212-2.440) | 0.596 |
| Thyroid diseases | 20 (17.7) | 13 (14.4) | 1.225 (0.557-2.698) | 0.614 |
| Anemia | 2 (1.8) | 4 (4.4) | 0..295 (0.046-1.869) | 0.195 |
| Uterine inertia | 0 | 3 (3.3) | - | 0.086^*^ |
| Abnormal amniotic fluid | 13 (11.5) | 9 (10.0) | 1.141 (0.452-2.880) | 0.732 |
| Puerperal infection | 6 (5.4) | 1 (1.1) | 4.635 (0.511-42.073) | 0.133^*^ |
| Apgar Score at one minute | 2 (1.8) | 1 (1.1) | 3.493 (0.108-112.817) | 1.000^*^ |
| Apgar Score at five minutes | 0 | 0 | - | - |

*Fisher exact test. #The generalized linear model was adjusted by the variables of age, BMI before pregnancy, parity and gravidity.
