# Supplementary material for: Effect of individualized weight management intervention on excessive gestational weight gain and perinatal outcomes: a randomized controlled trial
Source: PeerJ. 2022 Mar 8;10:e13067. doi: 10.7717/peerj.13067 (PMC8916027; doi:10.7717/peerj.13067)
Supplement: Supplemental Information 5 [file peerj-10-13067-s005.docx]

Supplemental Table 4. The comparison of weight gain of pregnancy women during pregnancy in the various BMI groups between the intervention and control groups

| **Group** | **Intervention** | **Control** | **Crude β (95%CI)** | **P** | **Adjusted β (95%CI)^*^** | **P** |
| --- | --- | --- | --- | --- | --- | --- |
| Underweight | 17.5±5.8 | 17.0±2.7 | 0.569 (-2.160, 3.299) | 0.683 | -0.17 (-2.935, 2.595) | 0.904 |
| Normal | 16.0±5.4 | 17.7±3.8 | -1.719 (-3.085, -0.353) | 0.014 | -1.633 (-3.018, -0.247) | 0.021 |
| Overweight | 15.4±5.3 | 17.7±3.8 | -2.271 (-4.392, -0.15) | 0.036 | -2.021 (-4.228, 0.186) | 0.073 |
| Obese | 13.0±5.2 | 17.2±3.6 | -4.128 (-7.667, -0.589) | 0.022 | -4.995 (-8.542, -1.448) | 0.006 |

*The variables of age, gravidity and parity were adjusted in the generalized linear model.
