# Supplementary material for: Effect of individualized weight management intervention on excessive gestational weight gain and perinatal outcomes: a randomized controlled trial
Source: PeerJ. 2022 Mar 8;10:e13067. doi: 10.7717/peerj.13067 (PMC8916027; doi:10.7717/peerj.13067)
Supplement: Supplemental Information 6 [file peerj-10-13067-s006.docx]

Supplemental Table 5. The comparison of weight gain of pregnancy women during pregnancy in the various BMI groups between during the second and third trimester groups

| **Group** | **Second trimester** | **Third trimester** | **Crude β (95%CI)** | **P** | **Adjusted β (95%CI)^*^** | **P** |
| --- | --- | --- | --- | --- | --- | --- |
| Underweight | 17.0±6.2 | 18.6±5.3 | -1.596 (-6.682, 3.491) | 0.539 | -0.886 (-6.268, 4.495) | 0.747 |
| Normal | 16.0±5.5 | 15.9±5.3 | 0.039 (-1.874, 1.951) | 0.968 | -0.08 (-2.018, 1.858) | 0.936 |
| Overweight | 14.9±5.6 | 15.8±5.0 | -0.912 (-4.082, 2.257) | 0.573 | -0.713 (-3.955, 2.529) | 0.666 |
| Obese | 11.7±4.4 | 20.5±0.7 | -8.818 (-15.09, -2.546) | 0.006 | -7.641 (-15.394, 0.111) | 0.053 |

*The variables of age, gravidity and parity were adjusted in the generalized linear model.
