# Supplementary figures and images for: Effect of individualized weight management intervention on excessive gestational weight gain and perinatal outcomes: a randomized controlled trial
Source: PeerJ. 2022 Mar 8;10:e13067. doi: 10.7717/peerj.13067 (PMC8916027; doi:10.7717/peerj.13067)

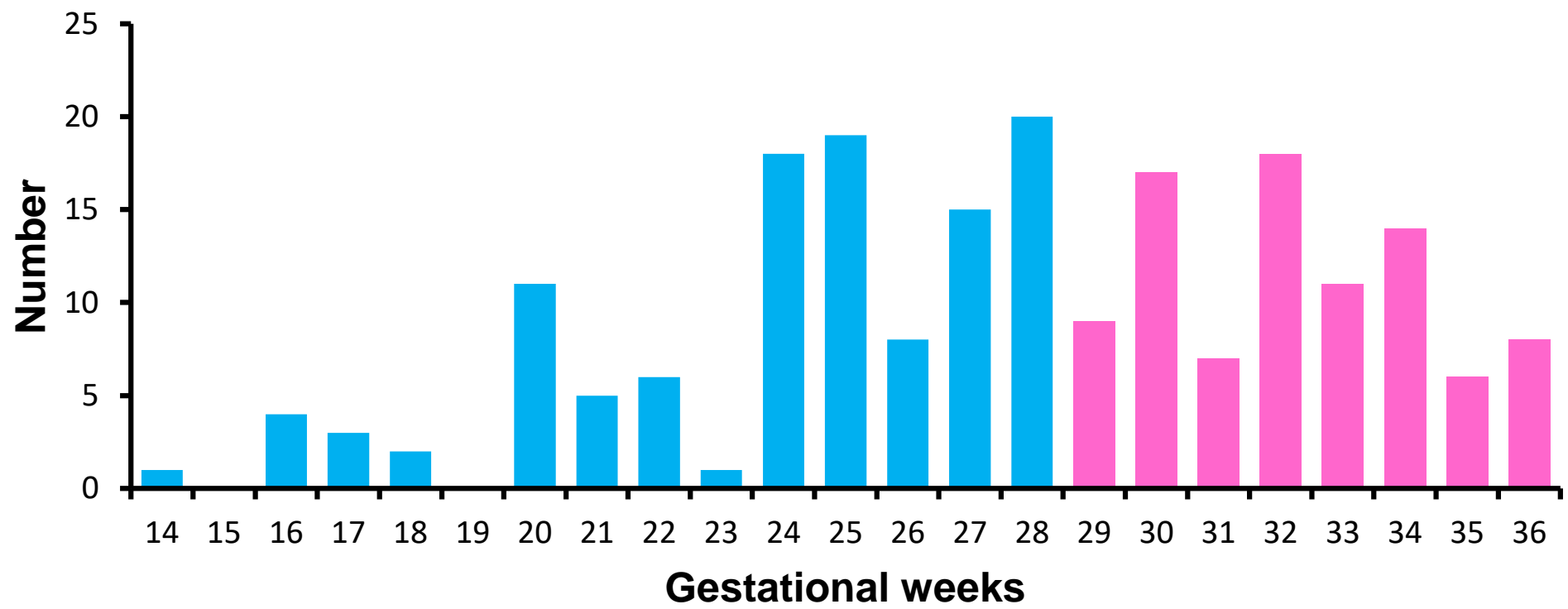

Supplement: Supplemental Information 7 — The gestational weeks of the pregnant women in the intervention group. The blue represents the second trimester, and the pink represents the third trimester. [file peerj-10-13067-s007.pdf]
