# Supplementary material for: Effect of individualized weight management intervention on excessive gestational weight gain and perinatal outcomes: a randomized controlled trial
Source: PeerJ. 2022 Mar 8;10:e13067. doi: 10.7717/peerj.13067 (PMC8916027; doi:10.7717/peerj.13067)
Supplement: Supplemental Information 8 [file peerj-10-13067-s008.doc]

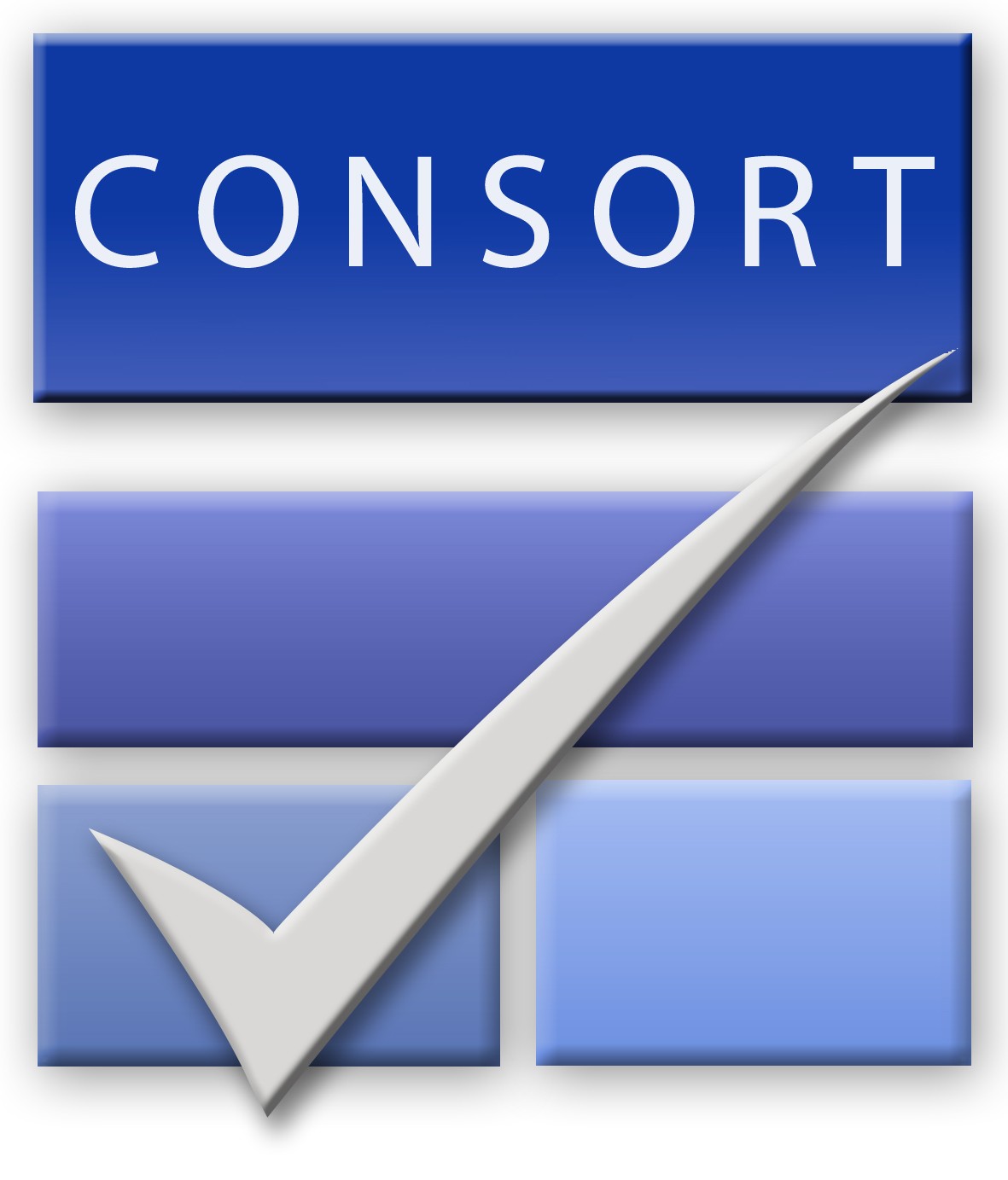
CONSORT 2010 checklist of information to include when reporting a randomised trial*

| Section/Topic | Item No | Checklist item | Reported on page No |
| --- | --- | --- | --- |
| Title and abstract | | | |
|  | 1a | Identification as a randomised trial in the title | Page 1 Line 1-2 |
|  | 1b | Structured summary of trial design, methods, results, and conclusions (for specific guidance see CONSORT for abstracts) | Page 2-3 Line 28-63 |
|  | | | |
| Background and objectives | 2a | Scientific background and explanation of rationale | Page 4-5 Line 73-118 |
|  | 2b | Specific objectives or hypotheses | Page 5 Line119-122 |
| Methods | | | |
| Trial design | 3a | Description of trial design (such as parallel, factorial) including allocation ratio | Page 5-6 Line 126-132 |
|  | 3b | Important changes to methods after trial commencement (such as eligibility criteria), with reasons | NA |
| Participants | 4a | Eligibility criteria for participants | Page 6 Line134-142 |
|  | 4b | Settings and locations where the data were collected | Page 5-6 Line 125-129 |
| Interventions | 5 | The interventions for each group with sufficient details to allow replication, including how and when they were actually administered | Page 6-8 Line 143-177 |
| Outcomes | 6a | Completely defined pre-specified primary and secondary outcome measures, including how and when they were assessed | Page 8 Line178-188 |
|  | 6b | Any changes to trial outcomes after the trial commenced, with reasons | NA |
| Sample size | 7a | How sample size was determined | Page 9 Line 200-208 |
|  | 7b | When applicable, explanation of any interim analyses and stopping guidelines | NA |
| Randomisation: |  |  |  |
| Sequence generation | 8a | Method used to generate the random allocation sequence | Page 9-10 Line 210-219 |
|  | 8b | Type of randomisation; details of any restriction (such as blocking and block size) | Page 9-10 Line 212-216 |
| Allocation concealment mechanism | 9 | Mechanism used to implement the random allocation sequence (such as sequentially numbered containers), describing any steps taken to conceal the sequence until interventions were assigned | Page 9 Line 210-212 |
| Implementation | 10 | Who generated the random allocation sequence, who enrolled participants, and who assigned participants to interventions | Page 9-10 Line 210-219 |
| Blinding | 11a | If done, who was blinded after assignment to interventions (for example, participants, care providers, those assessing outcomes) and how | Page 9-10 Line 217-219 |
|  | 11b | If relevant, description of the similarity of interventions | NA |
| Statistical methods | 12a | Statistical methods used to compare groups for primary and secondary outcomes | Page 10-11 Line 220-258 |
|  | 12b | Methods for additional analyses, such as subgroup analyses and adjusted analyses | Page 11 Line 244-252 |
| Results | | | |
| Participant flow (a diagram is strongly recommended) | 13a | For each group, the numbers of participants who were randomly assigned, received intended treatment, and were analysed for the primary outcome | Page 12 Line 261-269 |
|  | 13b | For each group, losses and exclusions after randomisation, together with reasons | Page 12 Line 262-264 |
| Recruitment | 14a | Dates defining the periods of recruitment and follow-up | Page 12 Line 262-263 |
|  | 14b | Why the trial ended or was stopped | Page 12 Line 263-264 |
| Baseline data | 15 | A table showing baseline demographic and clinical characteristics for each group | Page 12 Line 269 Table 1 |
| Numbers analysed | 16 | For each group, number of participants (denominator) included in each analysis and whether the analysis was by original assigned groups | Page 12 Line 262-269 |
| Outcomes and estimation | 17a | For each primary and secondary outcome, results for each group, and the estimated effect size and its precision (such as 95% confidence interval) | Page 12-14 Line 271-318 |
|  | 17b | For binary outcomes, presentation of both absolute and relative effect sizes is recommended | Page 12-14 Line 272-318 |
| Ancillary analyses | 18 | Results of any other analyses performed, including subgroup analyses and adjusted analyses, distinguishing pre-specified from exploratory | Page 13-14 Line 295-318 |
| Harms | 19 | All important harms or unintended effects in each group (for specific guidance see CONSORT for harms) | NA |
| Discussion | | | |
| Limitations | 20 | Trial limitations, addressing sources of potential bias, imprecision, and, if relevant, multiplicity of analyses | Page 17 Line 378-388 |
| Generalisability | 21 | Generalisability (external validity, applicability) of the trial findings | Page 14 Line 321-325 |
| Interpretation | 22 | Interpretation consistent with results, balancing benefits and harms, and considering other relevant evidence | Page 15-17 Line 326-377 |
| Other information | | |  |
| Registration | 23 | Registration number and name of trial registry | Page 6 Line 129-130 |
| Protocol | 24 | Where the full trial protocol can be accessed, if available | Page 6 Line 132-133 |
| Funding | 25 | Sources of funding and other support (such as supply of drugs), role of funders | Page 19 Line 425-432 |

*We strongly recommend reading this statement in conjunction with the CONSORT 2010 Explanation and Elaboration for important clarifications on all the items. If relevant, we also recommend reading CONSORT extensions for cluster randomised trials, non-inferiority and equivalence trials, non-pharmacological treatments, herbal interventions, and pragmatic trials. Additional extensions are forthcoming: for those and for up to date references relevant to this checklist, see [www.consort-statement.org](http://www.consort-statement.org/).
