# Supplementary material for: Effect of individualized weight management intervention on excessive gestational weight gain and perinatal outcomes: a randomized controlled trial
Source: PeerJ. 2022 Mar 8;10:e13067. doi: 10.7717/peerj.13067 (PMC8916027; doi:10.7717/peerj.13067)
Supplement: Supplemental Information 9 [file peerj-10-13067-s009.docx]

**Protocol**

**Title: Effect of individualized weight management intervention on excessive gestational weight gain and perinatal outcomes: a** **randomized controlled trial**

Research Institutions:

Department of Nutrition, Aerospace Center Hospital, Beijing 100049, PR China

Department of Obstetrics and Gynecology, Aerospace Center Hospital, Beijing 100049, P. R. China

Department of Laboratorial Science and Technology, School of Public Health, Peking University, Beijing 100191, P. R. China

**RESEARCH TEAM**

| **Protocol No.** | 201801 | **Version No.** | 1.0 |
| --- | --- | --- | --- |
| **Study Title** | **Effect of individualized weight management intervention on excessive gestational weight gain and perinatal outcomes: a randomized controlled trial** | | |
| **Leading Clinical Research Institution** | | | |
| Name: Department of Nutrition, Aerospace Center Hospital, Beijing 100049, PR China | | | |
| **Principal Investigator** | | | |
| Department of Obstetrics and Gynecology, Aerospace Center Hospital, Beijing 100049, P. R. China | | | |
| **Statistical Analysis** | | | |
| Institution name: School of Public Health, Peking University  Address: No.38 Xueyuan Road, Haidian District, Beijing  Contact: Qingbin Lu  Tel: 010-82805327  E-mail: qingbinlu@bjmu.edu.cn | | | |

# 1. STUDY OBJECTIVE

**1.1. Primary objective**

Evaluate the effect of individualized weight management intervention during the second and third trimesters for pregnancy women with excessive weight gain**.**

**2. TRIAL DESIGN**

**2.1 Design**

This trial uses a randomized design to evaluate the effect of individualized weight management intervention during the second and third trimesters for pregnancy women with excessive weight gain.

**Randomization**

Each subject was randomly placed into the intervention group or the control group according to the group result produced by a statistician using a computer in the clinic room. The randomization code was assigned to each participant in sequence in the order of enrolment, and then the participants were assigned to the group corresponding to that code. All eligible subjects were assigned a random number which was used to identify all procedures performed after the subjects had been randomly grouped. Once a random number had been assigned to one subject, it could not be reassigned to another subject. The intervention group was given an individualized weight management intervention without blinding method at the nutrition clinic of the Aerospace Center Hospital. We did not use the block randomization.

**Grouping**

Control group: standard dietary advice and precautions were given to the control groups by the doctors in the Department of Obstetrics and Gynecology. The target number of enrollments is 117.

Intervention group: standard dietary advice and precautions were given to the control groups by the doctors in the Department of Obstetrics and Gynecology, and individualized weight management intervention were given to build the study participants’ motivation, support, and self-efficacy for weight-related behavior change based on the Social Cognitive Theory. The target number of enrollments is 176.

**2.2 Prenatal examination data and pregnancy outcome**

The primary outcome measure was gestational weight gain during pregnancy, that was the difference between the weight on the day of delivery and the weight before pregnancy.

The secondary outcomes were the neonatal outcomes (including birth weight, asphyxia neonatorum, prematurity, fetal macrosomia, fetal distress and the Apgar score at one and five minutes) and the maternal outcomes (including delivery mode, GDM, premature rupture of membrane, postpartum hemorrhage, preeclampsia, gestational hypertension, thyroid diseases, anemia, uterine inertia, abnormal amniotic fluid, and puerperal infection), which were retrieved from the inpatient hospital records after delivery by the obstetrician.

We collected pre-pregnancy information by the doctors in obstetrics department which were self-reported by the pregnant women such as age, gravidity and parity, and the information at birth (the weight, gestation in weeks, and number of hospitalization days).

**2.3 Sample Size Estimation**

The sample size needed was determined to be at least 176 in the intervention group and 117 in the control group. A 95% power value was used to detect a difference of -2.0 between the null hypothesis that both groups’ means were 15.0 and the alternative hypothesis that the mean of control group was 17.0. The estimated group standard deviations were 5.0 and 4.0 for the intervention and control groups, which were estimated by the obstetrician and gynecologist based on previous data on the pregnant women, respectively, and the significance level (alpha) was set at 0.050 using a two-sided two-sample t-test.

**3. INCLUSION, EXCLUSION, TERMINATION, AND WITHDRAWAL CRITERIA**

**3.1 Inclusion Criteria**

The inclusion criteria were that pregnant women with excessive gestational weight gain according to the IOM guidelines were selected from outpatient services of the Aerospace Center Hospital; all participants were singleton pregnancy, no complications before pregnancy (i.e., type 2 diabetes mellitus, pre-pregnancy hypertension, and renal, immunologic, or hepatic diseases), signed the informed consent form, and all women intended to receive prenatal care and complete the pregnancy at our institution.

**3.2 Exclusion Criteria**

The exclusion criteria were that pregnancy outcome data were not available. All the participants were provided standard obstetrical care and nutrition education.

**4. Analysis sets**

**Full analysis set (FAS):**

The FAS is the ideal subject population determined based on the intention to treat (ITT) principle, which includes all randomized subjects.

**Per-protocol set (PPS):**

The PPS is a subset of the FAS that contains subjects with better protocol adherence. All subjects who conform to the inclusion/exclusion criteria, have completed all visits as required by the protocol, and were not removed are included in the PPS.

**5. Statistical analysis**

Descriptive statistics were performed, with continuous variables reported as the mean ± standard deviation (SD) for normal distribution and median (interquartile range, IQR) for abnormal distribution and categorical variables reported as frequencies and proportions. Generalized linear models assessed the association between the intervention or intervention time point and the continuous outcome variables, including the prenatal weight of pregnant women, weight gain during pregnancy, prenatal BMI, gain in BMI during pregnancy, newborn birth weight, gestational weeks and length of hospitalization. The β and 95% confidence interval (95%CI) were estimated. A logistic regression model was used to explore the rate difference of the categorical outcome variables (including caesarean, premature rupture of membrane, asphyxia neonatorum, prematurity, fetal macrosomia, GDM, postpartum haemorrhage, fetal distress, preeclampsia, gestational hypertension, thyroid diseases, anaemia, uterine inertia, abnormal amniotic fluid, puerperal infection, an Apgar score at one minute of < 10 and an Apgar score at five minutes of < 10) between the intervention and control groups or between the second and third trimester groups, which is when the participants began the individualized weight management plan. The risk ratio (RR) and 95% CI were estimated. We also analyzed the difference of weight gain between the intervention group and control group in the various of the pre-pregnancy BMI groups or in the second and third trimesters using the generalized linear models. For the two models above, the association was adjusted for age, pre-pregnancy BMI, parity and gravidity. The level of significance was set at 0.05. All statistical analyses were performed using Stata 14.0 (Stata Corp LP, College Station, TX, USA).

**6. Ethics approval and consent to participate**

This study was performed with the approval of the Ethical Committees of Aerospace Center Hospital. The methods were carried out according to approved guidelines. All study participants provided informed written consent prior to study enrollment.
